# Measurement of Internal Jugular Vein and Common Carotid Artery Diameter Ratio by Ultrasound to Estimate Central Venous Pressure

**DOI:** 10.7759/cureus.2277

**Published:** 2018-03-05

**Authors:** Sheher Bano, Aayesha Qadeer, Aftab Akhtar, Hafiz Muhammad Ata ur-Rehman, Kamran Munawar, Syed Waqar Hussain, Muhammad Tariq Khan, Rizwan Zafar

**Affiliations:** 1 Internal Medicine, Shifa International Hospital, Islamabad, PAK; 2 Critical Care, Shifa International Hospital, Islamabad, PAK; 3 Internal Medicine, Shifa College of Medicine, Islamabad, PAK; 4 Internal Medicine, Khan Research Laboratories Hospital, Islamabad, PAK; 5 Pulmonology, Shifa International Hospital, Islamabad, PAK; 6 Department of Internal Medicine, Shifa International Hospital, Islamabad, PAK

**Keywords:** central venous pressure, common carotid artery, internal jugular vein, volume status, critically ill patient

## Abstract

Objective

The objective of this study is to find a correlation between internal jugular vein (IJV) and common carotid artery (CCA) diameter ratio and central venous pressure (CVP) measurement and find a cut-off value for the IJV/CCA ratio to predict low CVP i.e. < 10 cm H_2_0, for estimating the volume status in critically ill patients.

Methods

This prospective cross-sectional study was conducted at the critical care department of Shifa International Hospital, Islamabad, from July to December 2017. A sample of 49 patients ≥ 18 years with intrathoracic central venous catheters (CVCs) who underwent bedside sonographic assessments of IJV and CCA diameter were included in this study using convenient sampling. The IJV/CCA diameter ratio was calculated and correlated with CVP and the predictive value of the IJV/CCA diameter ratio to predict CVP < 10 cm H_2_O was explored by calculating the area under the receiver operating characteristic (ROC) curve, sensitivity, specificity, and positive and negative predictive values.

Results

A total of 49 patients, 30 males (61.2%) and 19 females (38.8%) with a mean age of 56.00±16.11 years were included in the study. The mean CVP was 8.98±2.37cm H_2_O in ventilated (51%) and 10.7± 6.01 cm H_2_O in non-ventilated (49%) patients. The mean IJV/CCA diameter ratio was 1.60±0.55 at expiration and 1.41±0.56 at inspiration. There was a significant correlation between the IJV/CCA diameter ratio and CVP at expiration (r=0.401, p=0.004). The correlation between IJV/CCA and CVP was significant in non-ventilated patients at expiration (r=0.439, p=0.032). The area under the ROC curve for the IJV/CCA diameter ratio for predicting CVP < 10 cm H_2_O was 0.684 (p=0.028). The predictive value of the IJV/CCA diameter ratio for CVP < 10 cm H_2_0 at the cutoff value of < 2 was insignificant. A new cut-off < 1.75 was taken for the IJV/CCA diameter ratio from the coordinates of the ROC curve. The sensitivity, specificity, PPV, and NPV of an IJV/CCA diameter ratio of < 1.75 for predicting a CVP < 10 cm H_2_0 were 84.62%, 52.17%, 66.67%, and 75.00%, respectively.

Conclusion

The assessment of volume status by the IJV/CCA diameter ratio with a sonographic device may be a useful noninvasive alternative for a central venous catheterization with a cut-off < 1.75.

## Introduction

A bedside assessment of volume status is a crucial part of patient management in critically ill patients [1]. There are different invasive and noninvasive methods for volume assessment. The commonly used invasive parameters for volume assessment are pulmonary artery catheter (PAC) and central venous pressure (CVP). However, PAC use is becoming obsolete these days due to studies showing increased mortality with PAC placement [2]. More than 90% of intensivists use CVP to guide fluid management [3]. Some of the noninvasive modalities for volume status assessment include an ultrasonographic assessment of the inferior vena cava (IVC) collapsibility index, internal jugular or femoral vein collapsibility, and internal jugular vein/common carotid artery cross-section area [4-8].

Point of care ultrasonography is getting more importance these days in patient management, especially in emergency and intensive care units due to fewer complications [9]. Complications associated with central venous pressure (CVP) insertion include pneumothorax, bleeding and arterial punctures, the risk of local and distant infections, such as insertion site infections and catheter-related bloodstream infections, and septic emboli [4]. The IVC collapsibility index assessment by ultrasound is a convenient method for assessing volume status, but this modality is difficult to use in patients with recent abdominal surgeries, abdominal wounds, morbid obesity, and raised intra-abdominal pressure [5].

Recently, internal jugular vein (IJV) and common carotid artery (CCA) ratio measurement using ultrasound is found to be effective for CVP estimation. Najed et al. reported a significant positive correlation between the CVP and IJV/CCA ratio (r = 0.734, p < 0.05). Sensitivity, specificity, and the positive and negative predictive value of the IJV/CCA diameter ratio for CVP estimation were 90%, 86.36%, 90%, and 86.36%, respectively [6]. Hilbert et al. reported that the IJV/CCA ratio using ultrasound is an effective non-invasive alternative for CVP measurement among critically ill patients [10]. Baily et al. found that an IJV/CCA ratio ≥ 2 is significantly associated with a CVP > 8 mmHg (p < 0.05) [11].

To look for a reliable, noninvasive convenient method for volume status assessment in critically ill patients, we conducted this study in our non-western population, where we tried to find a correlation between the IJV/CCA diameter ratio and CVP measurement and to find a cut-off ratio for the IJV/CCA diameter ratio and low CVP i.e. < 10 cm H20.

## Materials and methods

This prospective cross-sectional study was conducted at the critical care department of Shifa International Hospital, Islamabad. The study was approved by the ethics and review board of Shifa International Hospital, Islamabad. It was conducted from July 2017 to December 2017. Informed consent was taken from the patients or their family members when patients were unable to consent for themselves. Patients were enrolled from the medical and surgical intensive care units. The inclusion criteria consisted of all patients ≥ 18 years of age admitted in the critical care area of the hospital and having an intrathoracic central venous catheter (CVC) in place for producing a CVP waveform through the transducer. Patients with pulmonary hypertension, any type of defect at the site of sonology, tricuspid regurgitation, inability to lay supine, instability of vital signs during sonography, and unwillingness to participate were excluded.

Sonographic assessments were performed by critical care fellows. They were formally trained and were given an orientation for the procedures protocol to minimize operator-related bias. It was followed by a practice examination under the critical care consultants and supervisors. The linear probe of the Mindray diagnostic ultrasound system, model Z6 (Mindray, South Carolina, USA) was used. Patients were placed in the supine position at the level of the bed with no pillow or other objects under the head. All measurements were obtained at the level of the cricoid cartilage. The first ultrasound gel was applied to the side of the neck. A vascular transducer was then placed lightly on the neck and manual pressure was used to compress the IJV, distinguishing it from the CCA. IJV measurements were performed both at the end of inspiration and of expiration.

CVP was measured immediately after the sonographic examination. It was measured manually using a pressure manometer from the distal lumen of the central venous catheter and a single-lead electrocardiogram strip. The zero point was taken at the level of the fourth intercostal space in the mid-axillary line to represent the level of the right atrium. CVP was measured from a recording at the end of expiration, with the patient supine. The sonographers were blinded to the CVP readings. Patients were divided into two groups based on a CVP cutoff point of 10 cm H2O (about 7 mmHg), which is the recommended threshold for predicting volume responsiveness [12-13].

Data analysis

The sample size was calculated as 49 using the World Health Organization (WHO) sample size calculator, taking the 5% level of significance, 80% power, mean value of IJV/CCA ratio 1.89, and SD 0.83 reported in the previous study by Nejad et al. [6].

All the data was entered and analyzed using SPSS version 21.0 (IBM, NY, USA). Results were presented as mean±SD for quantitative variables and relative frequencies (percentages) for categorical variables. The normal distribution of quantitative variables was assessed using the Shapiro–Wilk test and Q-Q plots. The independent t-test was used to investigate the probability of signiﬁcance of the differences of quantitative variables between two groups. The paired t-test and χ2 or Fisher’s exact test used for the assessment of the signiﬁcance of the differences of two quantitative variables and categorical variables, respectively. Correlations were done to test for linear relations between the IJV/CCA ratio and CVP by the Pearson correlation coefficient. Sensitivity, speciﬁcity, and positive and negative predictive values (PPV and NPV), as well as positive and negative likelihood ratios with 95% confidence interval (CI), were calculated. The diagnostic performance of the IJV/CCA diameter ratio was expressed as the area under the receiver operating characteristic (ROC) curve. A p value of < 0.05 was considered significant.

## Results

A total of 49 patients, 30 males (61.2%) and 19 females (38.8%), with a mean age of 56.00±16.11 years, were included in the study from July to December 2017. The mean CVP was 9.87±4.57 cm H_2_O (95% CI: 8.55 – 11.18). Among 49 patients, CVP was < 10 cm H_2_O in 26 (53.1%) and ≥ 10 cm H_2_O in 23 (47%). There were 25 (51%) ventilated and 24 (49%) non-ventilated patients. There was no significant difference in demographic data (Table 1).

**Table 1:** Patients’ characteristics CVP: central venous pressure, IJV: internal jugular vein, CCA: common carotid artery, SD: standard deviation, CI: confidence interval

Patients included, n	49	Mechanical ventilation Yes 25(51%)	Mechanical ventilation No 24 (49%)	p-value
Male, n (%)	30 (61.2%)	13 (52%)	17 (70.8%)	0.176
Female, n (%)	19 (38.8%)	12 (48%)	7 (29.2%)	
Age, mean ± SD (95% CI), years	56.00 ± 16.11	57.0 ± 13.58	54.96 ± 18.63	0.662
CVP, mean ± SD (95% CI), cm H_2_O	9.87 ± 4.57 (95% CI: 8.55 – 11.18)	8.98 ± 2.37	10.79 ± 6.01	0.169
IJV diameter expiratory, mean ± SD (95% CI), cm	1.15 ± 0.41 (95% CI: 1.029 – 1.267)	1.16 ± 0.36 (95% CI: 1.01 – 1.30)	1.14 ± 0.47 (95% CI: 0.94 – 1.33)	0.838
IJV diameter inspiratory, mean ± SD (95% CI), cm	1.1 ± 0.41 (95% CI: 0.89 – 1.13)	1.07 ± 0.41 (95% CI: 0.90 – 1.23)	0.96 ± 0.41 (95% CI: 0.78 – 1.13)	0.362
CCA diameter, mean ± SD (95% CI), cm	0.72 ± 0.10 (95%CI: 0.69 – 0.75)	0.73 ± 0.09 (95% CI: 0.69 – 0.78)	0.70 ± 0.11 (95% CI: 0.66 – 0.75)	0.316

Assessment of IJV

The mean IJV diameter was 1.15±0.41 cm (95% CI: 1.029 – 1.267) at expiration and 1.01±0.41 cm (95% CI: 0.89 – 1.13) at inspiration. There was no significant difference between ventilated and non-ventilated patients (p = 0.838 at expiration, p = 0.362 at inspiration) (Table 1). There was a significant correlation between IJV diameter and CVP (r = 0.472, n = 49, p-value = 0.001 at expiration and r = 0.348, n = 49, p = 0.014 at inspiration). The correlation was not significant in ventilated patients (r = 0.386, n = 25, p = 0.057 at expiration and r = 0.383, n = 25, p = 0.058 at inspiration). In non-ventilated patients, the association was significant (r = 0.544, n = 24, p = 0.006 at expiration and r = 0.434, n = 24, p = 0.034 at inspiration) (Table 2).

**Table 2:** Correlations between IJV diameter (at end expiration and end inspiration) and IJV/CCA (at end inspiration and end expiration) with CVP IJV: internal jugular vein, CCA: common carotid artery, CVP: central venous pressure

Correlation between	Total (49)		Mechanical ventilation Yes (25)		Mechanical ventilation No (24)	
	r-value	p-value	r-value	p-value	r-value	p-value
IJV (exp) with CVP	0.472	0.001	0.386	0.057	0.544	0.006
IJV (insp) with CVP	0.348	0.014	0.383	0.058	0.434	0.034
CCA with CVP	0.281	0.051	0.046	0.826	0.447	0.029
IJV (exp)/CCA with CVP	0.401	0.004	0.343	0.094	0.439	0.032
IJV (insp)/CCA with CVP	0.275	0.056	0.346	0.090	0.308	0.143

Assessment of CCA

The mean CCA diameter was 0.72±0.10 cm (95% CI: 0.69 – 0.75). There was no significant difference between ventilated and non-ventilated patients (p = 0.316) (Table 1). There was no significant correlation between CCA diameter and CVP (r = 0.281, n = 49, p = 0.051).

Assessment of IJV/CCA ratio

**Figure 1:**
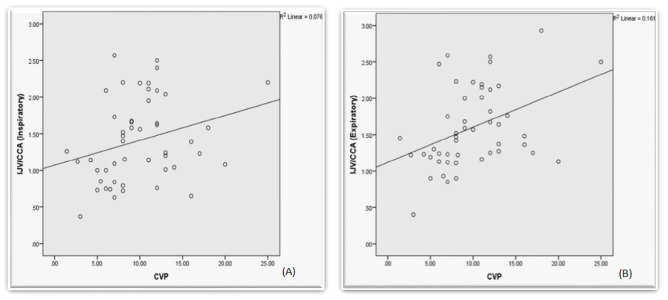
Relationship between central venous pressure (CVP) and internal jugular vein and common carotid artery (IJV/CCA) diameter ratio at inspiration (A) and at expiration (B).

The mean IJV/CCA diameter ratio was 1.60±0.55 (95% CI: 1.44-1.75) at expiration and 1.41±0.56 (95% CI: 1.24-1.57) at inspiration. There was a significant correlation between the IJV/CCA diameter ratio and CVP at expiration (r = 0.401, n = 49, p = 0.004) and a non-significant correlation at inspiration (r = 0.275, n = 49, p = 0.056) (Figure 1). The correlation between the IJV/CCA diameter ratio and CVP was non-significant in ventilated patients at both inspiration and expiration (r = 0.343, n = 25, p = 0.094 and r = 0.346, n = 25, p = 0.094, respectively). The correlation between the IJV/CCA diameter ratio and CVP was significant in non-ventilated patients at expiration (r = 0.439, n = 24, p = 0.032) and was non-significant at inspiration (r = 0.308, n = 24, p = 0.143) (Table 2).

The mean IJV/CCA ratio at expiration at CVP < 10 cm H_2_O was 1.39±0.50, and it was 1.83±0.52 at CVP ≥ 10 cm H_2_O with p = 0.004. The mean IJV/CCA ratio at inspiration at CVP < 10 cm H_2_O was 1.23±0.53, and it was 1.60±0.55 at CVP ≥ 10 cm H_2_O with p = 0.022 (Table 3).

**Table 3:** Comparison of the diameter of common carotid artery (CCA) and internal jugular vein (IJV) at inspiration and expiration between patients with a central venous pressure (CVP) < 10 or ≥ 10 cm H2O

	CVP < 10 cm H_2_O	CVP ≥ 10 cm H_2_O	p-value
	n = 26	n = 23	
CCA Diameter	0.70 ± 0.01	0.74 ± 0.11	0.173
IJV Diameter (exp)	0.97 ± 0.36	1.34 ± 0.39	0.001
IJV Diameter (insp)	0.87 ± 0.38	1.18 ± 0.39	0.007
IJV (exp)/CCA	1.39 ± 0.50	1.83 ± 0.52	0.004
IJV (insp)/CCA	1.23 ± 0.53	1.60 ± 0.55­­	0.022

**Figure 2:**
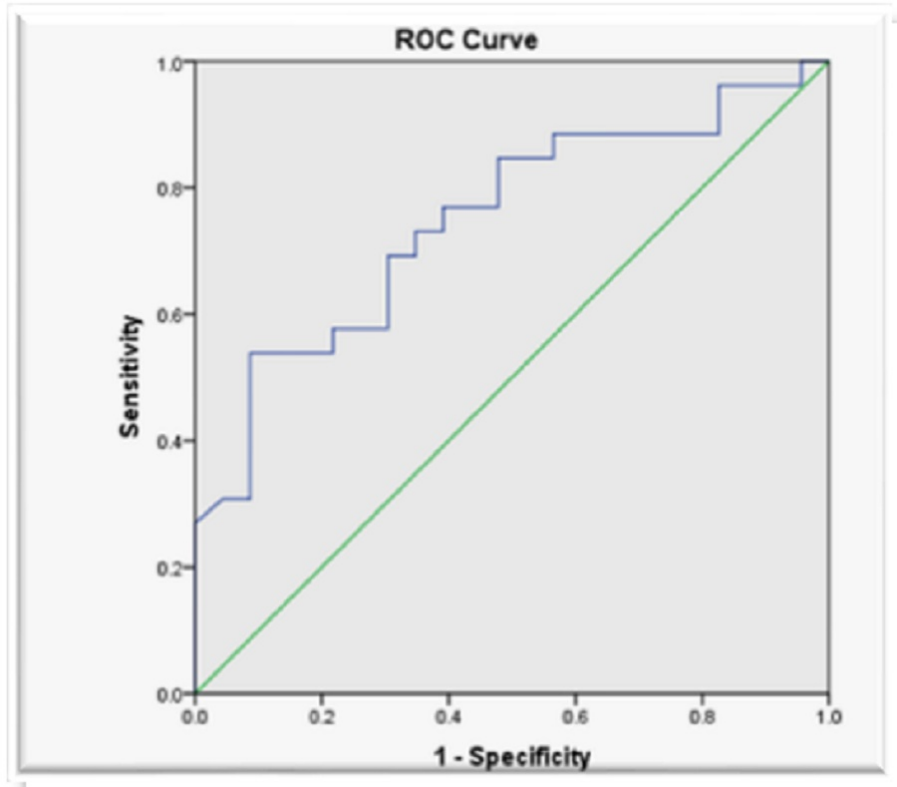
Receiver operating characteristic (ROC) curve for internal jugular vein/common carotid artery (IJV/CCA) ratio in predicting a central venous pressure (CVP ) < 10 cm H2O (area under the curve = 0.748)

The predictive value of the IJV/CCA diameter ratio for CVP < 10cm H2O at cutoff < 26 was found to be insignificant. The area under the ROC curve for the IJV/CCA diameter ratio was 0.640 (95% CI: 0.482 - 0.799), p-value > 0.05.The area under ROC curve for IJV/CCA diameter ratio for predicting CVP < 10 cm H2O was 0.748, p = 0.003 (Figure 2). A new cut off value < 1.75 was taken for IJV/CCA diameter ratio from the coordinates of the ROC curve. The sensitivity, specificity, PPV and NPV of an IJV/CCA diameter ratio of < 1.75 for predicting a CVP < 10cm H2O were 84.62%, 52.17%, 66.67% and 75.00% respectively. The positive likelihood ratio and the negative likelihood ratio were 1.77 and 0.29, respectively. The area under the ROC curve for the IJV/CCA diameter ratio for predicting CVP < 10 cm H^2^O was 0.684 (95% CI: 0.531-0.837, p = 0.028) (Table 4).

**Table 4:** Sensitivity, specificity, positive predictive value (PPV), negative predictive value (NPV), and positive and negative likelihood ratios of internal jugular vein/common carotid artery (IJV/CCA) for central venous pressure

	Central Venous Pressure < 10 cm H_2_O		
IJV/CCA Ratio <1.75	Positive	Negative	Total
Positive	22	12	34
Negative	4	11	15
Total	26	23	49
Sensitivity = 84.62%	PPV = 66.67%	Positive likelihood ratio = 1.77	
Specificity = 52.17%	NPV = 75.00%	Negative likelihood ratio = 0.29	

## Discussion

This study aimed to evaluate the IJV/CCA diameter ratio by ultrasound to estimate central venous pressure as a non-invasive tool for volume assessment. The current study showed that there is a moderate correlation of the IJV/CCA diameter ratio at expiration with CVP (r = 0.401), there is a poor correlation in patients who are on the ventilator (r =0.343). Cutoff < 2 for the IJV/CCA diameter, as reported by Nejad et al. [6], showed a significant result for predicting a CVP < 10 cm H_2_O. Our cut-off values of < 2 showed insignificant results (p>0.05); however, the new cutoff < 1.75 showed significant results (p = 0.028) with a sensitivity and specificity of 84.62% and 52.17%. The results reported by Nejad et al. [6] included patients who were non-ventilated and showed a strong correlation between the IJV/CCA ratio and CVP at expiration (r = 0.736) and at inspiration (r = 0.728), whereas our study showed a moderate correlation of the IJV/CCA diameter ratio at expiration with CVP (r = 0.439) in non-ventilated patients. So, a new cutoff for IJV/CCA < 1.75 is recommended, with significant results.

Another study that was conducted by Bailey et al. [11] reported that the IJV/CCA ratio could predict the value of CVP. It was a pilot study that was conducted in the pediatric population. Our population included adult intensive care patients, so results cannot be generalized.

IJV, as a measure of hemodynamic status, has also been evaluated. Donahue et al. [12] reported a significant difference in IJV diameter in patients with a CVP of < 10 or ≥ 10 cm H_2_O and a significant correlation of IJV end-expiratory diameter and CVP ( r = 0.82) was concluded. Our study reported a significant difference (0.97 versus 1.34 cm ) for IJV end-expiratory diameter in two groups and moderate correlation of IJV end-expiratory diameter and CVP (r = 0.544 ). Elsadek WM et al. [14] conducted a study on pediatric cardiac surgery patients to evaluate the IJV diameter and cross-sectional area to estimate CVP. It concluded a poor correlation as was in our study in patients who were on the ventilator.

IVC assessment is another non-invasive tool for the estimation of hemodynamic status. It has been studied widely and most accepted as the sonographic method of estimating CVP [8,15]. In comparison with the IJV/CCA ratio, the sonographic assessment of IVC is limited by difficulties in obese or surgical patients. Also, it needs extensive training. So, the IJV/CCA diameter ratio with a cutoff of < 1.75 can predict CVP in these patients with less technical difficulties.

Respiratory fluctuations affect the venous return to the heart [16]. Our study assessed the IJV/CCA diameter ratio versus CVP relationship both at end inspiration and end expiration. A significant difference was found in non-ventilated patients (p = 0.032 at the end of expiration and p = 0.143 at the end of inspiration). These findings are different from Nejad et al.'s [6] who concluded that the correlation between the IJV/CCA ratio and CVP is not affected by respiration.

Our study has a few limitations. First, it is a single-centered study, so its results may not be generalized. Second, because of its small sample size, it may have an imprecise estimation of results and may not be highly applicable to a wide variety of populations. Third, the sonographic measurements are usually operator-dependent, which may lead to an alteration of measurements and results.

## Conclusions

The assessment of volume status by the IJV/CCA diameter ratio with a sonographic device may be a useful, non-invasive alternative for a central venous catheterization with cutoff <1.75. However, further studies are needed to confirm the results of our study.
